# Resilience in the Face of Cancer: On the Importance of Defining and Studying Resilience as a Dynamic Process of Adaptation

**DOI:** 10.3390/curroncol31070297

**Published:** 2024-07-12

**Authors:** Melanie P. J. Schellekens, Laura C. Zwanenburg, Marije L. van der Lee

**Affiliations:** 1 Scientific Research Department, Helen Dowling Institute, Expert Centre for Psycho-Oncology, 3720 AB Bilthoven, The Netherlands; lzwanenburg@hdi.nl (L.C.Z.); mvanderlee@hdi.nl (M.L.v.d.L.); 2 Department of Medical and Clinical Psychology, Tilburg University School of Social and Behavioral Sciences, 5037 AB Tilburg, The Netherlands

**Keywords:** resilience, cancer, psychological distress, adaptive coping, social support, emotion regulation, prospective longitudinal designs, ecological momentary assessment, qualitative research

## Abstract

Resilience is defined as the maintenance or relatively quick recovery of mental health during and after adversity. Rather than focusing on psychopathology and its causes, resilience research aims to understand what protective mechanisms shield individuals against developing such disorders and translate these insights to improve psychosocial care. This resilience approach seems especially promising for the field of oncology because patients face stressor after stressor from diagnosis to survivorship. Helping patients to learn how they can best use the resources and abilities available to them can empower patients to handle subsequent stressors. In the past few decades, resilience has increasingly been considered as a dynamic process of adaptation. While researchers use this definition, resilience has not yet been studied as a dynamic process in the field of oncology. As a result, the potential of resilience research to gain insight into what helps protect cancer patients from developing psychopathology is limited. We discuss conceptual and methodological proposals to advance resilience research in oncology. Most importantly, we propose applying prospective longitudinal designs to capture the dynamic resilience process. By gaining insight in how cancer patients engage in protective factors, resilience research can come to its full potential and help prevent psychopathology.

## 1. Resilience in the Face of Cancer

A cancer diagnosis and its treatment have a major impact on patients’ lives and their significant others. Approximately 30 to 50% of cancer patients are at risk for developing mental disorders warranting psychological care [1,2]. At the same time, the majority of patients are not severely distressed and appear to be remarkably resilient in the face of cancer [3]. Resilience is defined as the maintenance or relatively quick recovery of mental health during and after exposure to adversity [4]. Studying resilience in the field of psychosocial oncology could help improve our understanding of what shields cancer patients from developing long-term heightened distress and psychopathology. Such protective factors (e.g., social support network, emotion regulation skills) could help patients to manage the shock of a cancer diagnosis and all the stressors that follow from it. By further strengthening these protective factors, resilience research provides the opportunity to support people at risk for mental disorders and, ultimately, prevent psychopathology among cancer patients.

Resilience is a complex construct that has sparked much debate among researchers [4,5,6]. In the past few decades, we witnessed how the understanding of resilience as a dynamic process of adaptation has gained traction [4,7]. That is, the maintenance or quick recovery of mental health is the result of a dynamic process of adaptation to an adverse life event or period of difficult life circumstances. Naturally, this implies that resilience should also be operationalized, measured and studied as a dynamic process of adaptation. Although more and more researchers use this definition, to our knowledge, resilience has not yet been studied as a dynamic adaptation process in the field of oncology [8,9,10,11,12,13,14]. As a result, the potential of resilience research to gain insight into what helps protect cancer patients from developing psychopathology is limited. In the present paper we aim to address this issue and discuss conceptual and methodological proposals to advance the resilience literature in the field of oncology.

## 2. The Potential of Resilience Research

Resilience research stems from the fact that despite being confronted with severe and even life-threatening challenges, many people show a pattern of temporary disturbance followed by a relatively rapid and successful recovery or a trajectory of undisturbed, stable mental health [15]. This is reflected in trajectory studies, showing how cancer patients either do not experience heightened distress shortly after diagnosis and maintain their mental health throughout the treatment and re-entry phase (i.e., maintenance of mental health) or become significantly distressed during diagnosis and recover during or shortly after active treatment (i.e., recovery of mental health) [16,17,18]. Resilience research aims to comprehend these differences; why some people do not develop mental health problems and others do, despite being subjected to seemingly similar stressors. Ultimately, it offers a paradigm shift away from disease-focused towards health-focused research [4,7], by investigating the mechanisms that can protect individuals against heightened distress and psychopathology. Resilience research entails a promising strategy that complements distress-focused research and can improve mental health.

This resilience approach seems especially promising for the field of oncology, as cancer patients face stressor after stressor. Diagnosis is often preceded by physical symptoms, medical procedures and great anxiety. Discovering that one has a life-threatening illness can be shocking and undermine one’s idea of invulnerability and sense of control. Anti-cancer treatment starts with processing complex medical information and decision making regarding different treatment options. Treatment is often invasive and can lead to medical complications and disabling side-effects (e.g., pain, fatigue), impairing one’s daily life, ability to work and social relations. Successful anti-cancer treatment is often followed up with recurring check-ups and fear of cancer recurrence or progression. Thus, a cancer diagnosis is not simply a major life event, it also marks a new life phase with significant chronic burden, in many cases extending months to years after cancer treatment has finished. On top of that, cancer patients become more sensitive to stressors in general. A cancer diagnosis and its treatment can be experienced as traumatic [19]. Traumatic stress can result in acute and chronic changes in neurochemical systems (cortisol and norepinephrine) and specific brain regions (amygdala, hippocampus and medial prefrontal cortex), which result in long-term changes in the brain circuits that are involved in the stress response [20]. As a result, traumatic stress narrows patients’ window of tolerance, which means that patients have less capacity to handle daily hassles and can experience such hassles as very distressing. Research has confirmed this idea and showed that while cancer patients report a similar number of daily life stressors compared to healthy controls, cancer patients tend to react more sensitively to these daily hassles (i.e., a larger drop in their mood after a stressor) [21]. By gaining insight into what helps patients manage all of these stressors following a cancer diagnosis, resilience research can inform us how to optimally support cancer patients. Helping patients to learn what resources and abilities (e.g., supportive community, adaptive coping skills) are available to them and how they can best use these for their own benefit, we can empower patients to handle subsequent stressors.

## 3. Defining Resilience as a Dynamic Process

One of the main problems of resilience research is that the term resilience has been defined in a variety of ways, making it difficult to measure and study the concept. Resilience has been defined both as an ability or capacity (e.g., the ability to bounce back from adversity), a predisposition (i.e., being resilient or not being resilient) and a dynamic process of adaptation [4,15,22]. Adding to the problem is that in the field of oncology, resilience has often been used as an overarching concept for positive personality characteristics (e.g., optimism, compassion), adaptive coping styles (e.g., illness acceptance, emotion regulation) and positive mental or physical outcomes (e.g., mental wellbeing, quality of life) [8,9,10,11,12,13,14,23]. Consequently, resilience has been studied as both a predictor, mediator and outcome, using a variety of questionnaires to assess resilience or related concepts. This diversity in definitions and operationalizations creates confusion on what resilience actually entails and limits the potential of resilience research to help us gain insight into what helps support cancer patients at risk for developing mental disorders.

As some people have protective factors readily available to them (e.g., having an optimistic predisposition or growing up in a supportive family), it comes more easily to them to bounce back after adversity. However, while it might be appealing to take a binary approach towards resilience, it is not something that individuals have or do not have [5]. In reality, resilience more likely exists on a continuum that may be present to varying levels in response to different types of adversity across different contexts (e.g., cancer diagnosis, divorce, work conflict).

**Figure 1:**
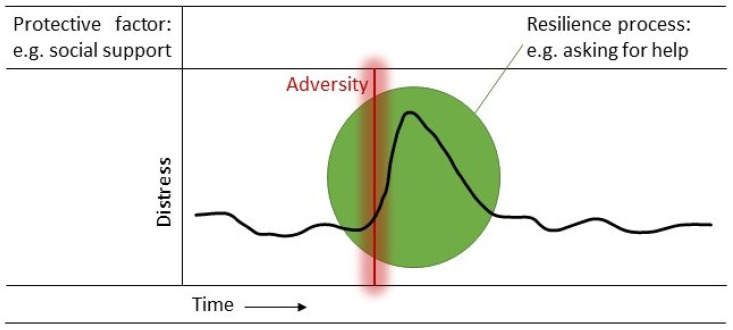
The dynamic process of resilience entails the engagement with a protective factor in response to adversity. For example, the individual draws upon their social support network (protective factor) by asking for help in response to a stressor (resilience process). A complex interplay of different protective factors, the type and intensity of the adversity and the context in which the individual resides determine to what extent the individual can engage in the available protective factors and respond resiliently.

In the past few decades, resilience is increasingly considered a dynamic process of adaptation [4,7]. Resilience is not simply a passive response or insensitivity to a stressor but the result of active, dynamic adaptation. But what is resilience then? What does this dynamic process of adaptation entail? Resilience researchers make a clear distinction between protective *factors* that contribute to the dynamic process of adaptation and the dynamic resilience *process* itself [7,24]. A protective factor represents more than simply a lack of risk; it includes predispositions, abilities and resources that are of benefit to one’s mental health. Predispositions and abilities are protective factors that reside within the individual, such as emotion regulation skills, an optimistic predisposition, mindfulness skills and self-efficacy, while resources are external protective factors and emphasize the influence of the social environment, such as support from family, friends and the community, peer support, and an adequate working alliance with healthcare professionals [25]. The resilience process itself entails the way in which individuals engage with these protective factors in response to a stressor [7,24]. For example, someone has certain emotion regulation skills (factor) and in response to a stressor they notice and accept their anxiety for what it is rather than trying to suppress it (process). Alternatively, the presence of a supportive family (factor) provides someone with the opportunity to request and accept help when confronted with one’s physical limitations (process). See Figure 1 for a depiction of the dynamic resilience process.

In daily life, there is not just one factor at play with which a person engages when facing adversity. Instead, it is a complex interplay of different protective factors at a behavioral, cognitive, emotional, physical, biological and/or social level in interaction with the type and intensity of the stressor, situated within a certain context, that determine to what extent the individual can engage with these protective factors and respond resiliently [26]. The interplay with the type and intensity of the stressor emphasizes the importance of studying resilience specifically in the context of cancer. The confrontation with cancer can be so overwhelming that it can completely knock people over. While it might have been relatively easy for the individual to use their resources in response to a stressor prior to cancer, it can be particularly difficult to manage stressor after stressor following a cancer diagnosis. Importantly, this interplay between protective factors and the adversity is always situated in a certain context, including one’s family, community, culture and society. Each of these contexts demonstrate more or less resilience of their own accord and, consequently, are capable of supporting the individual [27]. In a safe and stable community, it is easier for the individual to leverage certain community resources. Moreover, the level to which factors are experienced as protective is cultural and community specific [27]. For example, self-efficacy, attachment and ethnic identity might be considered generally important to resilience, but the relative importance of each depends on the community and culture in which the individual resides.

## 4. Informing Clinical Practice

When people suffer from a mental disorder, resilience appears diminished. However, this does not mean that cancer patients suffering from a mental disorder are non-resilient. The process view on resilience implies that a mental disorder is not a final state in which resilience is unachievable but rather a difficult and challenging condition where improving engagement with protective factors can play a key part in the road to recovery. In fact, it suggests that it is possible for anyone to learn to respond resiliently by engaging in the protective factors available to them.

This positive outlook allows resilience research to inform clinical practice [24]. First, by learning what protective factors help patients to respond resiliently in facing a cancer diagnosis and its treatment, we can strengthen these abilities and resources in patients who experience or are at risk for psychopathology. So far, a number of studies have demonstrated the effectiveness of resilience interventions that aim to strengthen such protective factors (e.g., emotion regulation skills) [28,29,30,31,32]. In the broader literature, positive psychology interventions have been found effective in enhancing well-being and reducing symptoms of depression [33,34]. These interventions supplement the more traditional ‘deficit-oriented’ psychosocial interventions by building on an individual’s strengths, resources and values (e.g., expressing hope, practicing gratitude, doing acts of kindness), and seem ideal candidates to help strengthen the protective factors that contribute to the individual’s ability to adapt to adversity. Recently, Amonoo and colleagues argued to extend distress screening during the cancer care trajectory with assessments of different aspects of well-being (e.g., hope, gratitude, positive affect) [35]. The goal of discussing these protective factors should not only be to identify those who need an intervention but also to uncover patients’ strengths and competencies that help them to respond resiliently to cancer treatment [35].

Second, by deepening our understanding of the way in which patients engage with these abilities and resources (i.e., resilience process) we can help them utilize these resources. As treatment (e.g., chemotherapy) and the traumatic stress caused by diagnosis and treatment can cause memory problems [20,36], patients sometimes fail to remember their own abilities and resources and how to engage with them. So, while protective factors might be present, there is no resilience. Thus, rather than simply strengthening protective factors (e.g., supportive friends), patients will likely benefit most from (re-)learning how to utilize these resources to their benefit in times of adversity (e.g., sharing concerns with friends) and in whichever context this will be most helpful to them [24,25]. If we want to gain insight into how and in what context patients can best engage in such protective factors, resilience needs to be studied as a dynamic process of adaptation.

## 5. Studying Resilience as a Dynamic Process

As resilience is not a static but dynamic concept, resilience should not be studied by itself but prospectively in response to a stressor. So far, however, the scoping, integrative and systematic reviews that have been published in the past decade on resilience in the face of cancer indicated that most studies assessed resilience cross-sectionally and after the adversity had occurred [9,11,12,13,14]. More specifically, resilience is often still operationalized as a score on one of the many available resilience questionnaires [37]. Assessing how someone generally handles adversity is problematic because it implicitly assumes that resilience is a stable characteristic that does not vary over time, across contexts or in interaction with different types of adversity. The longitudinal resilience studies that have been conducted have mainly focused on assessing participant’s health outcomes (e.g., levels of psychological distress) and retrospectively linking them to adversity (e.g., cancer diagnosis and cancer treatment) [11]. We advocate taking a prospective approach by observing adversity and directly assessing its influence on the individual [38]. Note that, despite using mainly post-adversity data, previous studies have offered valuable insight into how people can have happy and meaningful lives despite a cancer diagnosis and its treatment and identified important protective factors for these positive outcomes [3,11,16,17,18,39]. We do not argue that resilience can only be studied by using pre- and post-adversity data. However, we do want to emphasize that mapping mental health in the face of adversity prospectively offers a unique opportunity to capture the complexity of the dynamic resilience process. It allows us to move beyond the protective factors and identify the resilience process itself [38]. In other words, it allows us to study how a person draws upon their social support network (e.g., by sharing their concerns or asking for help) or how one uses their emotion regulation skills (e.g., by allowing one’s emotions or by distracting oneself) in reaction to a stressor.

So far, the reviews on resilience among adult cancer patients [9,11,12,13,14] that have been published in the past decade identified only a small number of studies that studied resilience by assessing mental health prior and post adversity. We reviewed these reviews and findings showed that four of the five reviews included studies using mostly cross-sectional designs that assessed resilience with a resilience questionnaire (see Table 1). By contrast, George and colleagues only included studies that assessed resilience using at least three assessment points [11]. They identified seven longitudinal studies that studied resilience by assessing mental health both prior to and post adversity (i.e., cancer diagnosis, cancer recurrence, radiotherapy and daily life stressors) [21,40,41,42,43,44,45]. These studies showed that the majority of patients showed a resilient trajectory (e.g., low depression scores from 2 years before to 4 years after diagnosis) [40]. While these studies identified a number of predictors of the resilient trajectory (e.g., better physical and mental health at baseline, cytokine gene variation and polygenic scores) [41,42,44,45], the resilience process itself was not examined.

We propose to use a quantitative longitudinal design in which mental health is assessed repeatedly prior to and after a stressor [7]. Below we argue why such a longitudinal design has the potential to capture the complexity of this dynamic process of adaptation. In addition, we propose a qualitative approach of patients’ experiential knowledge to gain insight into how they respond to adversity. Insight from qualitative research could offer valuable input for designing longitudinal studies.

### 5.1. Qualitative Approach

Seeking to understand how people see important experiences, qualitative research is an interpretive, naturalistic approach to peoples’ worlds, giving voice to their own experiences and perceptions [46]. Through qualitative interviews we can gain direct insight into what cancer patient do, think or feel in response to the challenges they face during cancer diagnosis, treatment and survivorship. We can explore what they experience as helpful (e.g., behaviors, mindsets, social interactions) in handling these stressful life circumstances. As such, it allows us to study the resources and abilities that are available to cancer patients (i.e., protective factors) and also how they engage with them (i.e., resilience process). For example, when participants mention they feel supported by their family and friends throughout cancer treatment, the interviewer can further explore what this looks like. What do participants do (e.g., seeking company, ask for help, share their concerns), at what moment (in times of distress or when they feel better), with whom, and what does the other person do (e.g., listen, give advice) that makes them feel supported? The interviewer can further explore whether this support is related to other protective factors. For example, does it take acceptance of one’s situation and trusting others before one is able to ask for help or share one’s concerns, or does social support help the individual to regulate one’s emotions better and to be more optimistic in times of distress? The qualitative approach also provides the opportunity to study the protective factors in relation to the stressor and the context in which the individual resides more elaborately. For example, the stressor of a cancer diagnosis has many aspects (e.g., new diagnosis or cancer recurrence, curative, life-prolonging or palliative treatment) that can determine its impact and, potentially, the interaction with protective factors and the resulting resilience process.

So far, qualitative research has mainly explored the difficulties cancer patients experience and less attention has been paid to what helps them handle these stressors [23,47,48]. Such hands-on practical knowledge of what patients experience as helpful could provide us with insight into the various resilience factors and processes that are at play in coping with cancer, e.g., [49,50]. Furthermore, it can inform us about the timeframe of the resilience process; the time it takes to recover from either major or everyday life stressors. Together, this information could offer valuable input for quantitative studies on resilience.

### 5.2. Longitudinal Designs

A prospective longitudinal design is ideally suited to study the dynamic resilience process. By assessing psychosocial functioning multiple times prior and after the stressor, we can assess whether distress was maintained or whether after initial reactivity there was a relatively quick recovery from distress [51]. Such a study design could be applied to a variety of stressors cancer patients experience: diagnosis, treatment or control scans. In the context of cancer, these adversities cannot be treated as isolated stressors because they are preceded and followed by other stressors. Therefore, it would be helpful to assess stressors and mental health problems repeatedly over a longer period of time. Note that next to mental health problems, positive outcomes can also be used to map the resilience process (see Box 1).

Box 1 **Positive emotions and their long-term benefits.**

There is a growing body of evidence that mental illness and mental health are, although correlated, independent concepts [52]. Key to mental illness and health are one’s emotions. Negative affect has an obvious adaptive value as it instantly narrows our thought–action repertoires to those that best promote survival in life-threatening situations. Negative emotions are associated with behavioral withdrawal and usually linked to short-term action tendencies [53]. For example, fear motivates the urge to escape and anger motivates the urge to attack. Although less obvious, positive emotions are also adaptive by helping individuals prepare for later hard times. The broaden-and-build theory posits that positive affect (joy, contentment, love) broadens an individual’s momentary mindset, which in turn helps to build enduring personal resources [54]. The literature shows that positive affect is characterized by behavioral approach, such as the urge to play, to explore new opportunities and to interact with others [53]. As such, positive emotions have more general and long-term functions by facilitating the development and strengthening of one’s abilities and resources, such as physical strength, social bonds, creativity and new cognitive skills. These resources can be drawn upon in subsequent moments of adversity, aiding the resilience process [54]. Research confirms this by showing that positive mental health protects cancer patients from developing psychopathology [55,56]. Thus, in line with the positive psychology movement, promoting positive adjustment, alongside alleviating vulnerabilities, will likely benefit patients long-term [57].

Next to major stressors, such as diagnosis and treatment, we should study daily hassles or so-called microstressors [58]. Such microstressors include, for example, feeling misunderstood by a friend, getting stuck in a traffic jam or feeling too fatigued to do the groceries. The reason these microstressors should be assessed is because these daily hassles are part of the chronic burden cancer poses on patients’ lives. As posited above, patients tend to experience such microstressors as more stressful than healthy controls [21]. Moreover, from trajectory studies we know that people who struggle with daily life stressors are more likely to develop psychopathology [59]. By zooming in on how patients manage these daily hassles, we can capture the resilience process in daily life.

Ecological Momentary Assessment (EMA) would be an ideal method to capture such microstressors. EMA is a structured diary technique, in which a participant receives questions multiple times a day for multiple days on end about their thoughts, feelings, activities and context in their daily living environment, usually via an app on their mobile phone [60]. EMA has often been used to assess microstressors, the intensity of the stressor, how one handled it and one’s mood [61,62]. This results in an intensive longitudinal dataset, making it possible to examine whether a microstressor is followed by a rapid recovery or maintenance of one’s mood. Resilience can be operationalized by relating the intensity of the stressor that occurred between the previous and present timepoint (i.e., adversity) to the change in mood between the previous and present timepoint (i.e., maintenance or rapid recovery of mental health) [63]. This provides several opportunities for resilience research. First, it allows insight into the dynamic resilience process. By exploring whether such resilient responses are influenced by certain baseline protective factors (e.g., social support network) and momentary items assessing to what extent the individual engages in this protective factor (i.e., sharing concern with significant other), we can unravel the dynamic resilience process in a detailed, ecologically valid manner at the microlevel. Second, it can be used to assess the effectiveness of resilience interventions. By including EMA as an outcome in a randomized controlled trial studying a psychological intervention that is supposed to improve the protective factors of resilience, we can assess whether people really improve in adapting to daily life stressors and what kind of protective factors they use during this adaptation process. This may offer strong evidence for such an intervention actually leading to improved resilience. Third, it can help identify at-risk individuals. Identifying those who have trouble adapting to daily life stressors helps us identify those patients who are likely to develop psychopathology and are in need of psychological treatment [59]. Fourth, it can provide input to help personalize treatment. The intensive longitudinal data provides the opportunity to go beyond group estimates and analyze processes within the individual. The protective factors the patient engages in or has trouble engaging in when confronted with daily life adversities can provide insight into what resources and abilities can be strengthened further. This offers valuable input for therapists to match treatment to the individual. Fifth, it has the potential to offer the individual patient the right support at the right time. As EMA monitors one’s ability to adapt to daily life stressors in real time, it can identify the moments when someone is struggling and might be in need of support. Underpinned by EMA and other mobile technologies, a just-in-time adaptive intervention (JITAI) aims to provide the right type and amount of support, at the right time, by adapting to an individual’s changing internal and contextual state [64]. Drawing from positive psychology interventions, offering a short gratitude exercise or a simple reminder to call a friend can be helpful micro-interventions to support the individual cancer patient when needed.

Despite concerns about the burden this method puts on patients, EMA has already shown promising first results concerning feasibility and usefulness in psychosocial oncology research [65]. Research suggests that as patients are actively involved in collecting data and gain insight into the personal, contextualized dynamics of their daily life, EMA can contribute to self-management and patient empowerment [66,67,68].

## 6. Conclusions

As resilience has been defined as a dynamic process of adaption to adversity, it should also be systematically studied as a dynamic process of adaption. Ultimately, this will reduce confusion in the field and misinterpretations by the public. By using intensive longitudinal designs, such as EMA, we can uncover what protective factors help patients to respond resiliently in the face of cancer and, more specifically, how patients engage in these factors during times of adversity. While this is much more time-consuming, energy-consuming and costly than cross-sectional studies using a resilience questionnaire, we are confident this will be worth it. By gaining insight in how patients engage in protective factors, resilience research can come to its full potential to inform clinical practice and, ultimately, help prevent psychopathology in those affected by cancer.

## Figures and Tables

**Table 1:** Reviews of resilience in adult cancer patients.

Review	Type of Review (Population)	Study Design	Questionnaire	Resilience Operationalized in Terms of Pre- and Post-Adversity Assessments of Mental Health
Eicher et al. (2015) [9]	Integrative literature review (mixed cancer)	Longitudinal (*n* = 4) Cross-sectional (*n* = 7)	Resilience (*n* = 11)	0/11
Lee et al. (2019) [13]	Scoping review (Korean, mixed cancer)	Pre-post intervention (*n* = 3) Cross-sectional (*n* = 14)	Resilience (*n* = 17)	0/17
Aizpurua-Perez et al. (2020) [14]	Systematic review (breast cancer)	Longitudinal (*n* = 1) Cross-sectional (*n* = 38)	Resilience (*n* = 39)	0/39
Sihvola et al. (2022) [12]	Systematic review (colorectal cancer)	RCT (*n* = 1) cross-sectional (*n* = 10)	Resilience (*n* = 11)	0/11
George et al. (2023) [11] *	Scoping literature review (mixed cancer)	Longitudinal (*n* = 17) (R)CT ** (*n* = 4) Pre-post intervention (*n* = 2) Qualitative (*n* = 3)	Resilience (*n* = 5) Mental health (*n* = 21)	7/26

* As 3 of the 29 studies focused on physical resilience, rather than psychological resilience, we report on the remaining 26 studies. ** (R)CT = randomised controlled trial and non-randomised controlled trial.
